# Deep Eutectic Solvents (DESs): Preliminary Results for Their Use Such as Biocides in the Building Cultural Heritage

**DOI:** 10.3390/ma15114005

**Published:** 2022-06-04

**Authors:** Andrea Macchia, Romina Strangis, Sara De Angelis, Marica Cersosimo, Antonella Docci, Michela Ricca, Bartolo Gabriele, Raffaella Mancuso, Mauro Francesco La Russa

**Affiliations:** 1 Department of Biology, Ecology and Earth Science (DiBEST), University of Calabria, 87036 Arcavacata di Rende, CS, Italy; andrea.macchia@uniroma1.it (A.M.); mlarussa@unical.it (M.F.L.R.); 2 YOCOCU (YOuth in COnservation of CUltural Heritage), Largo dei Quintili 21, 00175 Rome, Italy; saradeangelis05@gmail.com (S.D.A.); marica.cersosimo@gmail.com (M.C.); 3 Laboratory of Industrial and Synthetic Organic Chemistry (LISOC), Department of Chemistry and Chemical Technologies, University of Calabria, Via Pietro Bucci 12/C, 87036 Arcavacata di Rende, CS, Italy; romina.strangis@unical.it (R.S.); bartolo.gabriele@unical.it (B.G.); 4 Archaeological Park of Ostia Antica, Viale dei Romagnoli 717, 00119 Rome, Italy; antonella.docci@beniculturali.it

**Keywords:** DES, biocides, green conservation, cultural heritage, biodeterioration, solvent

## Abstract

Biodeterioration is an increasingly widespread process of degradation in the context of the conservation of cultural heritage, which involves a combination of physical and chemical damages together with an aesthetic alteration of materials. For biological damage on monuments caused by pathogens, macro- and microorganisms, chemical treatments are generally used, most of the time dangerous for the environment and for the operator. In this context, new eco-friendly products represent necessary tools for the treatment of biologically deteriorated stone surfaces and represent a new challenge in the field of restoration and conservation of materials of cultural interest. A relatively new class of unconventional green solvents are deep eutectic solvents (DESs), which have peculiar chemical-physical characteristics such as being non-toxic, ecological, biodegradable, non-flammable, and stable in the presence of water. Furthermore, many DESs known in the literature have also been shown to have a biocidal action. All these characteristics make DESs very advantageous and safe, and they could be used as biocidal agents for the treatment of biodegraded surfaces of cultural heritage, being non-toxic for the environment and for the operator. So far, they are used in various fields, but they still represent a novel frontier in the cultural heritage sector. The present research aims at testing five different DESs for the first time in cultural heritage. In particular, DESs are applied to a mosaic located in the Ostia Antica Archaeological Park (Rome), and their efficiency is compared with a biocide product currently used in the restoration field, namely, Preventol RI50, through luminescence, bio-luminometry, and spectrocolorimetry analysis. The preliminary results achieved show the different behaviors of each DESs, highlighting the possibility of employing them in the field of cultural heritage. Further studies have been planned, some of which are already underway, to investigate the properties of DESs and indicate any improvements to make them more effective, both as solvents and as biocides, and easy to apply to various types of materials. The results obtained from this first study are very promising for the use of DES as a new green strategy for cleaning and conservation treatments of materials in the field of cultural heritage.

## 1. Introduction

The problem of biological attack is increasingly widespread in the conservation of cultural heritage. This type of degradation is mainly found on works placed outdoors, but also indoors, and is due to the growth and accumulation of organisms, such as bacteria, algae, fungi, lichens, and mosses [1,2,3]. Depending on the levels of humidity, light, and nutrients, these organisms form complex and heterogeneous biostructures of biological patinas, which are called biofilms [4]. In this way, interactions between biofilm, substrate, and external environment are triggered, leading to the main biodeterioration processes, such as accumulation of dirt and particles, loss of material by contraction, and expansion, and so on.

The cleaning of surfaces from biodeterioration takes place through various mechanical, chemical, and physical systems so far. Generally, cleaning procedures include processes for removing or reducing layers that are deposited on the artwork over time, causing damage. The reason for their removal is due to the state of conservation of the material, which no longer adequately responds to its functionality or the aesthetic values, altering the perception of the artwork and sometimes causing irreparable damage [3,4,5].

The most used method to clean surfaces from biological colonization is based on chemical substances, exploiting the action of biocidal substances [4]. These techniques are characterized by being harmful to the operator and the environment [5,6,7,8,9].

In the field of conservation of cultural heritage, the biocides used are not numerous as they must have specific requirements such as efficacy at low concentrations towards target organisms, non-interference with the constituent material of the artwork, and low risks for humans and the environment. Among the major compounds used by restorers to prevent the spread of biological patinas on stone materials, there are biocides based on quaternary ammonium salts (QASs), such as Preventol RI50. Their biocidal action is based on the destabilization of the cell membrane structure, leading to rapid cell lysis [10]. Although QASs are used at maximum concentrations of 5%, showing good results as biocides in various fields, they are dangerous for the operator and the environment. Indeed, many studies observed an abundant presence of QASs in soils caused by their rapid absorption and strong resistance to biodegradation in anoxic/anaerobic conditions [11], as well as causing a reduction in the efficacy of biocides and an increased resistance to antibiotics in microbial communities over time [12].

Considering the aforementioned properties of commonly used products, there is an urgent need for new biocides for the cultural heritage field, which have a lower impact on the environment and the operator. In this regard, DESs (Deep Eutectic Solvents) shall be considered. DESs are non-toxic solvents having good biodegradability, high dissolution ability, non-flammability, chemical and thermal stability, low vapor pressure, low melting point, low-cost, and solvent-free preparation [13,14]. Generally, they are liquid at temperatures lower than 100 °C and can be prepared by mixing hydrogen bond donors (HBD) and acceptors (HBA) in a stoichiometric ratio [15]. The interactions between these components lead to a decrease in the melting point, caused by the delocalization of the charge through the hydrogen bond between the halide ion and the hydrogen donor part [15].

The most common components used for DESs were choline chloride (ChCl) or urea as HBA, and glucose or oxalic acid as HBD. Generally, DESs are identified as sustainable solvents displaying low toxicity. Indeed, they are eco-friendly compounds, with good stability in the presence of water, high biodegradability, and low toxicity [16,17,18].

Some mixtures of DESs also have a biocidal and inhibiting action against some bacterial strains, in particular, towards Gram-positives, which have a structure less complex than Gram-negatives and are therefore more easily attackable [16,19]. It should be considered that the antimicrobial properties of DESs depend on various factors, although the mechanisms are not yet clear. Indeed, in various studies, a different biocide response was found depending on the various components that make up the DESs, which is also different from the activity of the individual components. This could be due to the delocalization of charges that occurs during their formation. For example, one of the most commonly used salts in the preparation of DESs, ChCl, has a delocalized cation that favors greater interaction with the negative groups present on the surface of cell membranes, leading to distortion and ruptures of the cell wall. Therefore, it leads to increased toxicity against microorganisms. Another factor proposed to explain the toxicity of DESs is their acidity or alkalinity (pH). Indeed, it must be borne in mind that bacterial and fungal growth are favored by optimal pH ranges (6.5–7.5 and 5.0–9.0, respectively). At the same time, values that are far from optimal cause denaturation of the proteins found on the cell membrane of the microorganism, together with negative effects on cellular activity. Consequently, DESs with acidic pH will exhibit a more pronounced biocidal effect [16,17,18]. Moreover, in a few studies, some DESs have been used as antifungal products for eukaryotic microorganisms such as *Phanerochaete chrysosporium*, *Aspergillus niger*, *Lentinus tigrinus*, and *Candida cylindracea*, microorganisms also found on artifacts in the field of cultural heritage [16].

Due to these characteristics, DESs can be an alternative in the cultural heritage sector, as it is possible to combine and modulate them according to needs, varying the molar ratios of the components and the water content, thus enhancing their versatility.

For the first time, we propose, in this study, the use of specific DESs as new greener solvents with biocidal activity on a mosaic in the Archeological Park of Ostia Antica. DESs were evaluated and analyzed by luminescence, bioluminometry, and spectrocolorimetry in order to assess their biocidal capacity, comparing their performance with the results obtained from the application of Preventol RI50.

The DESs used in the present work (ChCl/Eg, ChCl/MalAc, ChCl/Gly, ChCl/OxAc, and ChCl/U) are synthesized from natural precursors that are present in nature, in plants, or as secondary metabolites in humans and animals. From studies reported in the literature, they appear to be environmentally friendly and safe for humans. For instance, choline chloride, which is the HBD component for all DESs studied, is a very safe chemical molecule. Indeed, choline, its precursor, is an essential substance for human beings [20]. Malonic acid is a compound found in natural biological systems, such as legumes, and plays an important role in the development of animal and human brains [21]. Oxalic acid and its salts are end products of the metabolism of many types of plants and are used in conjunction with ascorbic acid for their positive impact on delaying browning and maintaining the overall quality of litchi fruit [22]. Urea is a naturally occurring metabolite that is expelled by humans. Some studies have shown that it can inhibit the proliferation of tumor cells [23], and glycerol is a triol of natural origin too, widely used in pharmacology and, as it is well known, in the preparation of beauty products due to its high-power emollient. Etylen Glycol, on the other hand, is used as a raw material in the manufacture of polyester fibers and for antifreeze formulations, and in poly(ethy1ene glycols) (PEGS) for some pharmacological applications [24].

## 2. Materials and Methods

### 2.1. DESs Preparation

For the synthesis of DESs, Choline Chloride, Ethylene Glycol, Malonic Acid, Glycerol, Oxalic Acid, and Urea were purchased from Sigma Aldrich (Merck KGaA, Darmstadt, Germany).

DESs were synthesized by weighing and mixing, at the proper molar ratio, the HBD and HBA molecules and by mixing and heating them (80–100 °C) until homogeneous liquids were formed in times spanning from 1 to 3 h. The chemical structures of solvents’ components and their molar ratios are reported in Table 1. The pH value of each DES is also reported in Table 1.

DESs, as already mentioned, are a eutectic mixture, which occurs in the liquid state following the union of two solid components. The DESs’ pH is due to the solvation water, present in very little amounts, which is closely anchored to the DESs. The nature of DESs is such that the two components, even if acidic, cannot release the proton, because they are strictly interconnected by an intrinsic donor-acceptor relationship. Only in aqueous solution, with a dilution greater than 50% (*v*/*v*) [25], would the eutectic nature, of the mixture, be destroyed and the acid component of DESs would be given the possibility to act as an acid species. Since DESs are used pure in the present work, and not in aqueous solution, they cannot release protons and therefore cannot interact with the stone layer but can only carry out antibacterial activity on the biofilm.

### 2.2. Experimental Set and Analytical Methods

**Figure 1:**
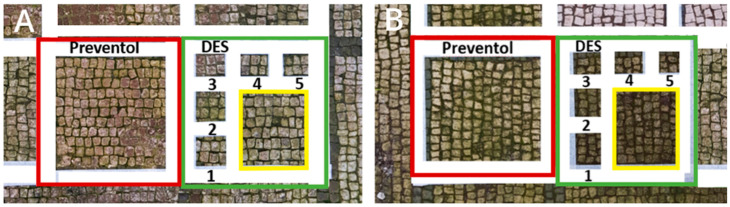
Two selected sample-areas of the mosaic in the Archeological Park of Ostia Antica: Area 1 (**A**) and Area 2 (**B**). The areas provided for the treatment with DESs (code 1–5) have been indicated with the green box, the one with Preventol Rl50 in red, and the untreated area in yellow.

Two areas of a mosaic flooring in the Archeological Park of Ostia Antica were selected as sample areas to conduct the experimentation (Area 1, Area 2), further subdivided into nine zones. In this study, only two zones (Figure 1) related to the application of Preventol RI50 (framed in red) and DESs (framed in green) will be considered. One area was left untreated and used as a reference (framed in yellow).

The products were applied by brush, creating an even layer. DESs were used “pure”, without diluting them in organic solvents or water. The experimentation was conducted in September 2021 with the following microclimate: 24 ± 2 °C and 61 ± 5% of UR%. They were left to act for 3 days, and then they were removed with a mechanical action by brushing and washing.

A bioluminometer (KAIROSafe PD30) was used to determine the biological contamination of the surfaces treated with DES products and Preventol RI50. For this purpose, was used the wipe method, repeating the test three times which allowed subsequent statistical analysis [26]. The analysis is based on the phenomenon of bioluminescence, which is the ability of living organisms to emit light through enzymatic reactions, converting chemical energy into light energy. More specifically, this phenomenon is determined by the reaction that occurs between the luciferase enzyme, the luciferin substrate, and the adenosine triphosphate (ATP) molecule, used by animal and plant cells to accumulate, and exchange energy. Consequently, the amount of light emitted will be directly proportional to the amount of ATP present in the sample, expressed by the Relative Light Units value (RLU), thus providing an assessment of the overall levels of organic understanding of the surface under consideration. It should consider that damaged cells or dead cells will no longer be able to produce ATP.

To investigate the distribution of biofilms on the surface before and after treatment, ultraviolet fluorescence imaging was acquired using a Madatec multispectral imaging system, in order to capture the visible light emitted by the microorganisms still present after the application of the studied biocides.

The evaluation of the chromatic variation was observed using a spectrocolorimeter (Y3060 3nh, Shenzhen 3nh Technology Co., Ltd., Shenzhen, China) in the CIE L* a* b* space, where the L* coordinate represents the brightness, a* and b* represent the chromaticity coordinates, on the green/red axis and on the blue/yellow axis, respectively. Then, chromatic alterations were analyzed by studying the ∆E, defined by the following equation:

$$\Delta E=\sqrt{{\left(\Delta L^{*}\right)}^{2}+{\left(\Delta a^{*}\right)}^{2}+{\left(\Delta b^{*}\right)}^{2}}$$

given the differences between the chromatic parameters of the surface before and after the treatment (∆L*, ∆a*, ∆b*). These measurements allow us to monitor bacterial growth on the surface under examination, as negative ∆L* and ∆a* and positive ∆b* values indicate a bacterial recolonization process, while for ∆L* and ∆a* values positive and negative ∆b* there will be bacterial decolonization of the surface, as well as biocidal efficacy of the analyzed solvents [27]. The measurements were repeated three times to reduce the uncertainty of the analysis.

The interaction between DESs and the substrate (mosaic tesserae) was studied in laboratory through electrical conductivity measurements using a low porosity stone (marble samples) and the most soluble DES used in the experimentation, as follows: Choline-Chloride:Urea (DES 5). Soluble salts are the main degradation problem in porous stone materials, due to the formation of efflorescence (sub- and surface-).

Electrical conductivity measurements were carried out to study the release of chlorine ions in the stone substrate, thus assessing the solvent’s interference with the constituent materials of the artwork. A HD2156.2-Delta OHM (Delta OHM S.r.l., Caselle di Selvazzano, Italy) was used for analysis. In total, 700 mg of DES was applied to the marble sample with a steel spatula. The marble sample was placed in an Angelantoni climate chamber for 24 h at the following different temperatures: 20, 40, 60 °C, and 70% U.R. These parameters were defined based on the temperature and humidity of Ostia Antica during the experimentation time as follows: 24 ± 2 °C and 61 ± 5% of UR%.

After 24 h the DES was removed from the marble sample. The marble sample was left in 400 mL of deionized water (EC = 7 ± 2 μS/cm) for 3 h. The electrical conductivity was measured and referred to the concentration of totally dissolved DES in 400 mL of deionized water (750 ± 5.6 μS/cm). The test was repeated three times to reduce the uncertainty of the analysis.

## 3. Results and Discussion

From ultraviolet fluorescence analysis (Table 2), it is possible to distinguish the distribution of the microorganisms present in the two areas of the pavement through the light that is re-emitted by the chlorophyll molecules constituting the microorganisms when these are affected by UV radiation. In the two areas, before treatment, red fluorescence is observed, which is faded after the application of the biocides, following the devitalization of the photosynthetic microorganisms.

In Area 1, a progressive biocidal response is observed for DES 5, 4, and 2, followed by DES 3 and 1; in Area 2, for DES 4, 2, and 5, followed by DES 3 and 1. This response is in agreement with the literature [28]. Indeed, DES 2 and 4, consisting of malonic acid and oxalic acid, respectively, have a more acidic pH, therefore creating conditions that limit bacterial growth.

Despite the good performance obtained, DES does not reach the standards obtained by using Preventol RI50 (Köln, Germany).

Anyway, it is extremely important to underline that the DESs studied in the present work, compared to Preventol RL50, commonly used in the field of cultural heritage, are stable, non-volatile, environmentally friendly, and do not require solvents for their dilution. Indeed, they can be applied pure because they are liquids or slightly viscous at room temperature. DESs are characterized by the mixing of two natural components that are not harmful to humans and therefore to the operator who applies the product to the stone artifact.

**Figure 2:**
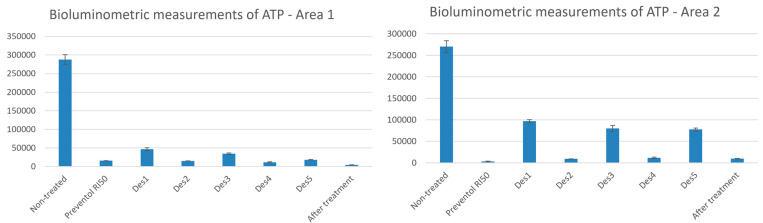
Bioluminometric measurements of ATP of the areas of interest. The values of RLU and SD are reported for Area 1 and Area 2 comparing them to the non-treated and the mechanically and chemically treated areas.

The bioluminometric measurements for Area 1 and Area 2 are shown in Figure 2. The values of RLU and standard deviation (SD) are reported, indicating the average and the SD of the different areas, comparing the non-treated and treated areas. Consequently, a good biocidal action is represented by low values reported in Figure 2, indicating a lower production of ATP due to the death or damage of the cells of the microorganisms capable of producing it.

From this, the best results were obtained for Area 1 and Area 2 by DES 2, DES 4, and by Preventol RI50, which show the lowest residual biological activity.

Table 3 shows the variations of the colorimetric parameters (L*, a*, b*) after the application and removal by mechanical cleaning of the biocides tested in the two areas, calculated as the geometric distance that separates them from the colorimetric parameters L*, a*, b* of the same areas before treatment.

The average values of the color parameters L*, a*, b* before treatment in Area 1 correspond to L* 41.01; a* 2.56; b* 19.87; for Area 2, there are average values of L* 45.87; a* 0.78; b* 23.61. For Area 1, positive ∆L* values and negative ∆a* values are observed for each area treated by the DES, while for Preventol Rl50, there is a negative ∆L* value (−3.96) and ∆a* positive (2.05). As regards ∆b*, there are negative values, except for Preventol Rl50 (0.15). This indicates that the areas following the treatment show a greening (∆a* negative), although not perceptible as the decrease in terms of absolute value is minimal. The increase in brightness can be a consequence of the removal of the biological patina, which allows the color of the mosaic tiles to re-emerge. In contrast to Area 1, Area 2 shows negative ∆L* values for most DES, except for DES 5 (6.62) and Preventol Rl50 (0.18). This decrease, although minimal, causes a slight graying of the surface. Positive and negative ∆a* and ∆b* values are observed for each treated area, indicating less greening and yellowing. Therefore, it is possible to state that the best results are obtained from the application of DES 2 and 4 for Area 1, presenting an increase in the L* parameter and a decrease in the a* and b* parameters; of DES 5 for Area 2 since ∆L* and ∆a* increase, while parameter b* decreases, thus leading to less yellowing.

**Figure 3:**
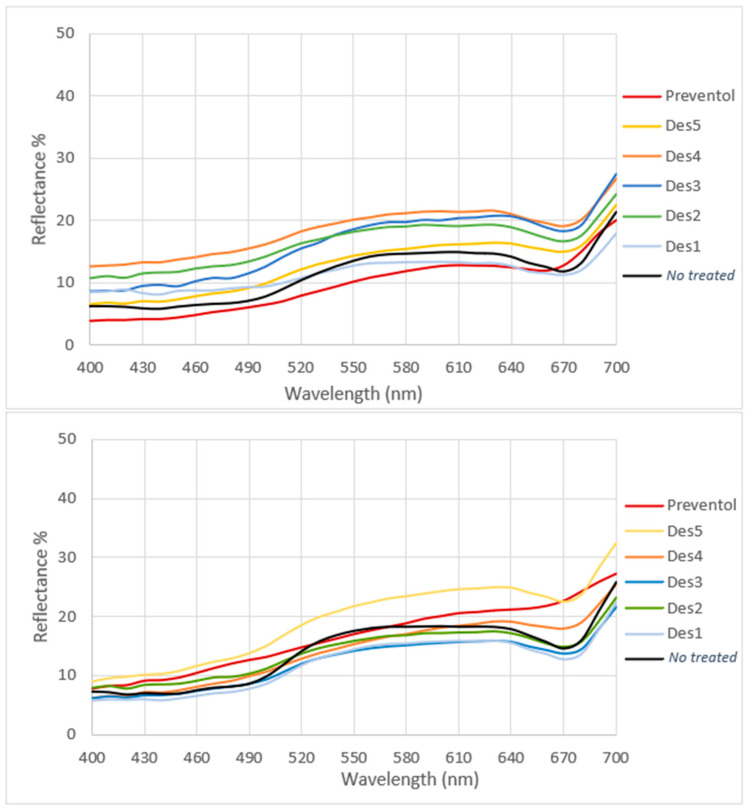
Full-spectrum color measurement of the treated zone in Area 1 (**above**) and in Area 2 (**below**) in comparison with the no treated zone indicated in black.

Figure 3 shows the full spectrum color measurements of the treated and untreated areas (indicated by a black curve). From the spectra, it is possible to observe an absorption of around 670 nm, relating to the absorption of chlorophyll, for most of the biocides applied. However, this trend is especially decreased in both the areas where Preventol T150 was applied and, to a lesser extent, in the areas treated with DES 2 and 5 (Area 1), indicating the reduction of living microorganisms. Indeed, photosynthetic biodeteriogens lose chlorophyll when they die—together with the green color—thus enabling the evaluation of the treatment’s efficacy by examining the typical absorption at 670 nm induced by ultraviolet light (UV) at 365 nm. A reduction in absorption reflects a reduction in microorganisms’ vitality.

Electrical conductivity tests allowed us to define the capillary absorption of the DES by the porosity of marble using the ratio between the EC measured on the DES residue present in the sample and the EC of the totally dissolved DES in water and the relative error (expressed in %). A 100% ratio means that all the amount of DES was absorbed by the sample. The results can be seen in Table 4.

The test defined a low capillary absorption of DES by the substrate in the microclimatic conditions that characterized the archaeological site of Ostia Antica during the experimentation. The absorption of chlorine by the stone substrate increases as a function of the temperature due to the deliquescence of the salts used as precursors for the formation of the DES.

## 4. Conclusions

Currently, the interest in DESs is increasing due to their specific characteristics, especially for their simple preparation, low cost, and versatility in various fields. In this study, for the first time, their biocidal action in the field of cultural heritage was tested as an alternative to traditional biocides, which are more dangerous for the environment and the operator. Five DESs with different compositions based on choline chloride with ethylene glycol, malonic acid, glycerol, oxalic acid, and urea were tested and compared with the traditional biocide Preventol RL50. The latter was applied in-situ on a mosaic in the Ostia Antica Archaeological Park. The treatment involved the application of the biocidal products (a water solution of Preventol RL50 and pure DESs) and the removal of the biological patina by mechanical action. To evaluate the efficacy of the products and the best treatment with DESs, the following various analytical investigations were used: ULV imaging, spectrocolorimeter, and bioluminometry. From the analysis carried out, the best results among the deep eutectic solvents studied were obtained by DES 2, 4, and 5, although their biocidal action is lower than that of the traditional Preventol Rl50. It was possible to observe a greater biocidal effect for acid-based DESs (malonic acid = DES 2; oxalic acid = DES 4) as they have a pH that does not fall within the ranges favorable to bacterial growth. The DES 5 (ChCl/U) showed good efficacy at a more basic pH (7.2 ± 0.5). DES studied in the present work, compared to Preventol RL50, commonly used in the field of cultural heritage, are stable, non-volatile, environmentally friendly, and do not require solvents for their dilution because they can be applied pure because they are liquids and slightly viscous at room temperature. DESs are characterized by the mixing of two natural components that are not harmful to humans and, therefore, to the operator who applies the product to the stone artifact. Following the recent introduction of these green solvents in the field of cultural heritage, their effectiveness over time has not yet been clarified, but from the preliminary data obtained in the present work, they could be promising and very performing in the field of cultural heritage. Future experiments can therefore be oriented in this direction.

## Figures and Tables

**Table 1:** Composition and pH of DES studied in this paper.

DES Code	DES Composition	HBD	HBA	Molar Ratio (HBD:HBA)	pH
DES 1	ChCl/Eg	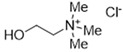 Choline Chloride	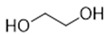 Ethylene glycol	1:2	5.6 ± 0.5
DES 2	ChCl/MalAc	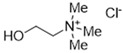 Choline Chloride	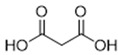 Malonic Acid	1:1	2.8 ± 0.5
DES 3	ChCl/Gly	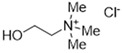 Choline Chloride	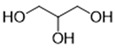 Glycerol	1:2	5.3 ± 0.5
DES 4	ChCl/OxAc	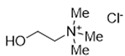 Choline Chloride	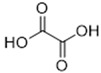 Oxalic Acid	1:1	3.2 ± 0.5
DES 5	ChCl/U	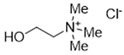 Choline Chloride	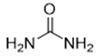 Urea	1:2	7.2 ± 0.5

**Table 2:** Ultraviolet fluorescence imaging of Areas 1 and 2, before and after treatment with DESs and Preventol RI50.

	Area 1		Area 2	
	Preventol RI50	DES	Preventol RI50	DES
Before the treatment	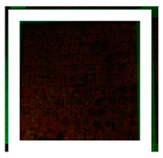	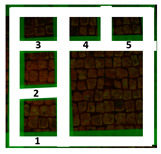	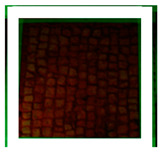	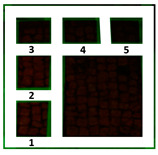
After the treatment	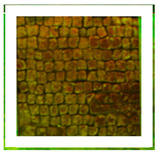	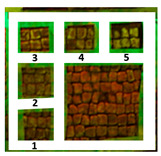	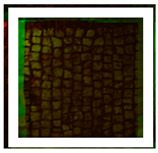	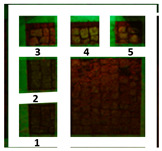

**Table 3:** Colorimetric parameters (∆L*, ∆a*, ∆b*, and ∆E) of Area 1 and Area 2.

	Area 1				Area 2			
Biocides	∆L*	∆a*	∆b*	∆E	∆L*	∆a*	∆b*	∆E
Preventol Rl50	−3.96	2.05	0.15	4.46	0.18	2.78	−6.17	6.77
DES 1	0.15	−1.13	−9.63	9.70	−4.01	0.96	−2.64	4.89
DES 2	6.78	−1.97	−7.52	10.32	−0.29	0.55	−6.96	6.99
DES 3	5.03	−0.67	−1.81	5.39	−2.89	0.88	−4.93	5.78
DES 4	9.37	−1.52	−6.75	11.65	−0.63	2.85	−3.86	4.84
DES 5	1.69	−0.34	−1.73	2.44	6.62	1.40	−2.17	7.10

**Table 4:** Results obtained from electrical conductivity measurements (EC) on laboratory samples treated with Choline-Chloride:Urea.

Amount of Applied DES (mg)	Temperature (°C)	Amount of Residual DES in the Specimens (mg)	EC (μS/cm)	Corresponding DES in Water (%)
523 ± 5	60	304 ± 2	414 ± 6.4	55%
512 ± 7	40	55 ± 2	73 ± 5.7	9%
520 ± 4	25	34 ± 5	41 ± 5.3	6%
